# An empirical analysis of post-work grocery shopping activity duration using modified accelerated failure time model to differentiate time-dependent and time-independent covariates

**DOI:** 10.1371/journal.pone.0207810

**Published:** 2018-11-21

**Authors:** Ke Wang, Xin Ye, Jie Ma

**Affiliations:** Key Laboratory of Road and Traffic Engineering of Ministry of Education, College of Transportation Engineering, Tongji University, Shanghai, China; University of Texas School of Public Health, UNITED STATES

## Abstract

In this paper, the accelerated failure time (AFT) model is modified to analyze post-work grocery shopping activity duration. Much previous shopping duration analysis was conducted using the proportional hazard (PH) modeling approach. Once the proportionality assumption was violated, the traditional accelerated failure time (TAFT) model was usually selected as an alternative modeling approach. However, a TAFT model only has covariates with non-proportional and time-dependent effects on the hazard overtime while a PH model only accommodates covariates with proportional and time-independent effects. Neither of them considers the possibility that some of covariates may have proportional and time-independent effects and some may have non-proportional and time-dependent effects on the hazard value in one model. To address this issue, the paper generalizes the TAFT model and develops a modified accelerated failure time (MAFT) model to accommodate both time-dependent and time-independent covariates for activity duration analysis. Checking on the proportionality assumption indicates that the assumption is not valid in the post-work grocery shopping activity data extracted from the 2017 National Household Travel Survey (NHTS) conducted by the U.S. Department of Transportation (USDOT). Both TAFT and MAFT models are developed for comparisons and analysis. The empirical and statistical results show that there do exist two different types of covariates affecting shopping activity duration, including covariates only with proportional and time-independent effects (i.e. working duration, commute travel time) and those with non-proportional and time-dependent effects. The MAFT model can capture the subtleties in various types of covariate effects and help better understand how those covariates affect activity duration overtime. This paper also shows the importance to develop a flexible duration model with both time-dependent and time-independent covariates for accurately evaluating travel demand management (TDM) policies, like flexible work hours.

## 1. Introduction

The shift from trip-based to activity-based models of travel demand has resulted in a focus on the analysis of activity-travel patterns [1]. Activity-travel patterns include many dimensions, such as the timing of activity, duration of activity, location of activity, mode of activity, and activity sequencing [2]. Modeling one of these dimensions helps to understand relevant decision process of other dimensions. Non-work activity duration, an important dimension of activity-travel patterns of individuals, greatly influences timing of travel and peak-period congestion. Non-work trips, which are derived from non-work activities, account for 53.5% of US residents’ daily trips [3]. Since the individuals have more flexibility to pursue non-work activities, congestion control policies or other traffic control measures may have more significant impacts on flexible non-work trips than on rigid commute trips. Among these non-work trips, travel for shopping takes a considerable portion of travel demand and significantly influences traffic congestion in urban areas [4]. Exploring shopping activity will help planners and researchers to find the determinants of shopping activity duration and take measures to reduce travel demand generated by shopping activity, and much research effort has been made in this regard. These studies include the departure time of urban shopping activities [5,6], duration of shopping activities and its determinants [2,4,7], and frequency of individuals’ shopping activity participation [8–10].

Many researchers have focused on the duration of shopping activities, an important dimension of activities. The factors influencing shopping activity duration initially aroused interest of researchers. Bhat (1996a) analyzed individual shopping activity duration on commute way and found that an individual’s shopping duration is affected by the individual’s work characteristics, spouse’s work characteristics, mode to work, and whether the individual is a young adult or not [2]. Sreela *et al*. (2013) modeled the shopping duration of employed individuals and identified some influential factors including household characteristics, individual characteristics and activity-travel variables [4].

Most research on duration phenomena in transportation is carried out using the hazard-based duration model. The hazard-based duration model, which was initially developed to model duration phenomena in biometrics and econometrics, has been extensively applied in a number of transportation fields including activity duration [2,4,7,11–13], traffic incident duration [14–17], driving behavior [18–23], and so on. Hensher and Mannering (1994), Bhat (2001), Bhat *et al*. (2004), Rashidi and Mohammadian (2011) provided extensive overviews of duration model applications in transportation [24–27]. The PH (Proportional Hazard) model is the most widely applied method based on the assumption that the hazard ratio of two individuals does not vary over time. Once the proportionality assumption is violated, an AFT (Accelerated Failure Time) model usually can be selected as an alternative modeling approach. Bhat (1996a) used a proportional hazard based model with nonparametric baseline and nonparametric heterogeneity to model the individual shopping activity duration on the way home from work [2]. Sreela *et al*. (2013) applied both parametric and semi-parametric (Cox) hazard-based approaches to model the duration of shopping activity and finally a parametric model based on Weibull distribution without heterogeneity was selected due to its better performance [4]. When the proportionality assumption of the PH model was violated, Karimi *et al*. (2013) used a latent segmentation AFT model to study the inter-shopping duration of seniors and non-seniors [9]. However, a traditional AFT (TAFT) model only has covariates with non-proportional and time-dependent effects on the hazard overtime while a PH model only accommodates covariates with proportional and time-independent effects. Proportionality is achieved by having a baseline hazard that does not depend on the covariates. Therefore, a covariate having “proportional” effect on the hazard function must be time-independent and able to scale up the baseline hazard function; a covariate with “non-proportional” effect on the hazard is time-dependent or interacts with time in the baseline hazard function. The effect of a time-dependent covariate on the hazard varies over time but the effect of a time-independent covariate is a constant over time. Proportional or non-proportional, time-dependent or time-independent, two different types of covariate effects on the hazard, can form 4 (2×2) possible combinations. Neither of PH and TAFT models can allow some covariates to have proportional and time-independent effects, some to have non-proportional and time-dependent effects on the hazard in one model. In this study, a MAFT (Modified AFT) model is developed by generalizing the TAFT model to address this issue and then applied to model post-work grocery shopping activity duration in Texas, US.

The remainder of this paper is organized as follows. Section 2 generalizes the TAFT model and develops the MAFT model to differentiate time-dependent and time-independent covariates for activity duration analysis. Section 3 provides an overview of the data source and the sample description. Section 4 presents the empirical model estimation results. Finally, Section 5 provides conclusions and discussions.

## 2. Methodology

### 2.1 The TAFT model

Hazard-based duration model is to study the conditional probability that a duration ends at some time *T*, given that the duration has continued until time *t*. Let *T* be a non-negative random variable representing the activity duration, and *t* is a specific time in the continuous time scale. Then the hazard function at time *t* can be defined as:

$$h(t)=\lim_{\Delta t\to 0}\frac{P(t\leq T\leq t+\Delta t)}{\Delta t}=\frac{f(t)}{S(t)}$$ (1)

where *h(t)* is the conditional probability that an activity will end between time *t* and *t*+Δ*t* given that the activity has not ended up to time *t*; *f(t)* is a failure density function; and *S(t)* is a survival function representing the probability that the activity still remains until time *t*.

The traditional AFT (TAFT) model assumes that covariates interact with time directly in the baseline hazard function, *h*_0_(*t*). Assume that *e*^−***Xβ***^ is the functional form of covariates’ effects, and then the hazard function at time *t* can be formulated as:

$$h(t|X)=h_{0}(t∙e^{-X\beta }){∙e}^{-X\beta }$$ (2)

where ***X*** and ***β*** are vectors of covariates and their coefficients, respectively. And the effects of covariates ***X*** on the hazard in Eq (2) are time-dependent and non-proportional at the same time. Note that the effect of a covariate is formulated by incorporating a negative sign for each coefficient. A negative sign allows a covariate with a positive coefficient to decease the hazard value but increase duration when the covariate increases.

Once either one formula among the hazard function, cumulative hazard function [denoted as *H(t)*], survival function, failure function [denoted as *F(t)*], or failure density function *f(t)* is known, the other four formulas of the TAFT model can be derived and all the relevant formulas are summarized as below:

$$H(t|X)=\int_{0}^{t}h(T|X)\;dT=\int_{0}^{t}h_{0}(T{∙e}^{-X\beta }){∙e}^{-X\beta }\;dT=H_{0}(t∙e^{-X\beta })=-\ln\left[1-F_{0}(t{∙e}^{-X\beta })\right]$$ (3)

$$S(t|X)=\exp\left[-H(t|X)\right]=\exp\left[-H_{0}(t{∙e}^{-X\beta })\right]=S_{0}(t{∙e}^{-X\beta })=1-F_{0}(t{∙e}^{-X\beta })$$ (4)

$$F(t|X)=1-S(t|X)={1-S}_{0}(t{∙e}^{-X\beta })=F_{0}(t{∙e}^{-X\beta })$$ (5)

$$f(t|X)=h(t|X)∙S(t|X)=h_{0}(t{∙e}^{-X\beta }){∙e}^{-X\beta }∙S_{0}(t{∙e}^{-X\beta })=f_{0}(t∙e^{-X\beta }){∙e}^{-X\beta }$$ (6)

where *H*_0_(*t*), *S*_0_(*t*), *f*_0_(t) and *F*_0_(*t*) are respectively baseline cumulative hazard, survival, failure density and failure functions associated with the baseline hazard *h*_0_(*t*). And the hazard function can also be transformed into a formula represented by *f*_0_(*t*) and *F*_0_(*t*):

$$h(t|X)=\frac{f(t|X)}{S(t|X)}=e^{-X\beta }{∙f}_{0}(t{∙e}^{-X\beta })/[1-F_{0}(t{∙e}^{-X\beta })]$$ (7)

### 2.2 The MAFT model

Considering the fact that not all the covariates accelerate or decelerate time directly in the baseline hazard, a TAFT model can be modified to develop a MAFT model. Assume that the vector of covariates ***W*** interacts with time in the baseline hazard and the vector of covariates ***Z*** exerts a proportional effect on the hazard function, then a modified hazard function can be written as:

$$h(t|W,Z)=h_{0}(t∙e^{-W\eta }){∙e}^{-Z\gamma }$$ (8)

where ***η*** and ***γ*** are respectively the vector of coefficients for covariates ***W*** and ***Z***.

Then the cumulative hazard function can be shown as:

$$H(t|W,Z)=\int_{0}^{t}h(T|W,Z)\;dT=\int_{0}^{t}h_{0}(T∙e^{-W\eta }){∙e}^{-Z\gamma }\;dT=e^{-Z\gamma }∙\int_{0}^{t}h_{0}(T∙e^{-W\eta })\;dT=\frac{e^{-Z\gamma }}{e^{-W\eta }}∙\int_{0}^{t}h_{0}(T∙e^{-W\eta })\;d(T∙e^{-W\eta })=\exp\left(W\eta -Z\gamma \right){∙H}_{0}(t∙e^{-W\eta })$$ (9)

Covariates in the vector ***W*** have non-proportional and time-dependent effect on the hazard function, covariates in the vector ***Z*** have proportional and time-independent effect on the hazard. It is possible that the same covariate occurs in both vectors of covariates ***W*** and ***Z***. There may be four different situations for one covariate. 1) A covariate may only occur in the vector ***Z*** and has a proportional and time-independent effect on the hazard value, which is identical to a covariate in the PH model. 2) A covariate can simultaneously appear in the vector ***W*** and ***Z*** with equal coefficients, which is same as a covariate in the TAFT model and has non-proportional and time-dependent effect on the hazard. 3) A covariate only exists in the vector ***W***, it directly interacts with time in the baseline hazard without additional effect on the hazard function; in other words, it has non-proportional and time-dependent effect on the hazard function. 4) Finally, a covariate may exist in both vectors of ***W*** and ***Z*** with unequal coefficients. Since simulation experiments show that unequal coefficients in the fourth situation are weakly identified [28], there are only three situations remaining in practice.

In order to ensure that the MAFT model has a reasonable interpretation, it is necessary to check whether a positive coefficient of an explanatory variable can monotonically increase the duration or decrease the hazard. An explanatory variable *x*_1_ in the first situation is the same as that in a PH model:

$$h(t|x_{1})=h_{0}(t|x_{1})e^{-x_{1}\beta_{1}}$$ (10)

$$H(t|x_{1})=H_{0}(t|x_{1})e^{-x_{1}\beta_{1}}$$ (11)

$$\frac{dH(t|x_{1})}{dx_{1}}=-\beta_{1}e^{-x_{1}\beta_{1}}H_{0}(t|x_{1})$$ (12)

where *H*_0_(∙) is an increasing function that takes a value greater than 0. When *β*_1_>0, $\frac{dH(t|x_{1})}{dx_{1}}<0$, indicating that when the covariate *x*_1_ increases, the hazard decreases and the duration increases.

A covariate *x*_2_ in the second situation is the same as that in a TAFT model:

$$h(t|x_{2})=h_{0}(t\;e^{-x_{2}\beta_{2}})e^{-x_{2}\beta_{2}}$$ (13)

$$H(t|x_{2})=H_{0}(t{\;e}^{-x_{2}\beta_{2}})$$ (14)

$$\frac{dH(t|x_{2})}{dx_{2}}=-\beta_{2}\;t\;e^{-x_{2}\beta_{2}}\;{H_{0}}^{\prime }(te^{-x_{2}\beta_{2}})$$ (15)

Since *H*_0_(∙) is an increasing function, *H*_0_′(∙) is greater than 0. When *β*_2_>0, $\frac{dH(t|x_{2})}{dx_{2}}<0$, indicating that when the covariate increases, the hazard decreases and the duration increases.

For an explanatory variable *x*_3_ in the third situation:

$$h(t|x_{3})=h_{0}(t\;e^{-x_{3}\beta_{3}})$$ (16)

$$H(t|x_{3})=e^{x_{3}\beta_{3}}{∙H}_{0}(t\;e^{-x_{3}\beta_{3}})$$ (17)

$$\frac{dH(t|x_{3})}{dx_{3}}=\beta_{3}e^{x_{3}\beta_{3}}[H_{0}(te^{-x_{3}\beta_{3}})-t\;e^{-x_{3}\beta_{3}}\;{H_{0}}^{\prime }(t\;e^{-x_{3}\beta_{3}})]$$ (18)

No matter whether *β*_3_ is greater than 0 or less than 0, when $t=\frac{H_{0}(te^{-x_{3}\beta_{3}})}{e^{-x_{3}\beta_{3}}{H_{0}}^{\prime }(te^{-x_{3}\beta_{3}})},\;\frac{dH(t|x_{3})}{dx_{3}}=0$. Thus, with the increase of *t*, the sign of $\frac{dH(t|x_{3})}{dx_{3}}$ may change. The time when the sign changes depends on the form of *H*_0_(∙) function. It means that *x*_3_ with a positive coefficient does not always increase the duration when it increases, which results in a challege to interpret the sign of a variable coefficient. Thus, the third situation will not be considered in a MAFT model.

Then, the final hazard function in a MAFT model can be summarized as:

$$h(t|U,V)=h_{0}(t∙e^{-V\alpha }){∙e}^{-V\alpha -U\theta }$$ (19)

where ***U*** is a vector of covariates in the first situation, ***V*** is a vector of covariates in the second situation, ***θ*** and ***α*** are respectively the vector of coefficients for covariates ***U*** and ***V***. It indicates that covariates in ***U*** all have proportional and time-independent effects on the hazard, and covariates in ***V*** all have non-proportional and time-dependent effects on the hazard.

Based on the hazard function, the other four functions of the final MAFT model can be shown as:

$$H(t|U,V)=\int_{0}^{t}h(T|U,V)\;dT=\int_{0}^{t}h_{0}(t∙e^{-V\alpha }){∙e}^{-V\alpha -U\theta }\;dT=e^{-U\theta }∙\int_{0}^{t}h_{0}(T∙e^{-V\alpha })\;d(T∙e^{-V\alpha })=e^{-U\theta }{∙H}_{0}(t∙e^{-V\alpha })=-e^{-\mathrm{U\theta}}∙\ln\left[A\right]$$ (20)

$$S(t|U,V)=\exp\left[-H(t|U,V)\right]=\exp\left[-e^{-U\theta }{∙H}_{0}(t∙e^{-V\alpha })\right]={\left[S_{0}(t∙e^{-V\alpha })\right]}^{\exp\left(-U\theta \right)}=A^{\exp\left(-U\theta \right)}$$ (21)

$$F(t|U,V)=1-S(t|U,V)=1-{\left[S_{0}(t∙e^{-V\alpha })\right]}^{\exp\left(-U\theta \right)}=1-A^{\exp\left(-U\theta \right)}$$ (22)

$$f(t|U,V)=h(t|U,V)∙S(t|U,V)=h_{0}(t∙e^{-V\alpha }){∙e}^{-V\alpha -U\theta }∙{\left[S_{0}(t∙e^{-V\alpha })\right]}^{\exp\left(-U\theta \right)}=e^{-V\alpha -U\theta }{∙f}_{0}(t∙e^{-V\alpha })∙{\left[S_{0}(t∙e^{-V\alpha })\right]}^{\exp\left(-U\theta \right)-1}=e^{-V\alpha -U\theta }{∙f}_{0}(t∙e^{-V\alpha })∙A^{\exp\left(-U\theta \right)-1}$$ (23)

where A = 1−*F*_0_[*t* ∙ *e*^−***Vα***^]. And the hazard function can also be transformed into a formula represented by *f*_0_(*t*) and *F*_0_(*t*):

$$h(t|U,V)=\frac{f(t|U,V)}{S(t|U,V)}=e^{-V\alpha -U\theta }{∙f}_{0}(t∙e^{-V\alpha })/A$$ (24)

Alternative parametric distributions are available for the AFT model with different shapes of hazard function. The exponential AFT has a constant hazard and the Weibull AFT has a monotonic hazard. The log-logistic, lognormal, Gamma and Gompertz distributions assume a non-monotonic hazard. The Weibull is the only distribution that can be applied under both accelerated life and proportional hazard models [29], and the exponential is a special case of Weibull distribution when the shape parameter equals 1. In practice, alternative distributions need to be compared based on goodness-of-fit measures and the one with the best performance can be recommended.

### 2.3 Model estimation

The above models can be estimated by using the standard maximum likelihood estimation (MLE) method. The likelihood function for the MAFT model is:

$$L=\prod_{i=1}^{N}{\left[f(T_{i}|U_{i},V_{i})\right]}^{1-M_{i}}∙{\left[S(T_{i}|U_{i},V_{i})\right]}^{M_{i}}$$ (25)

where *N* is the sample size, *T*_*i*_ is the survival time of observation *i*. *M*_*i*_ = 0 if *T*_*i*_ is not censored and 1 otherwise. Right censoring can occur when the activity hasn’t ended at the time when the observation is censored. For the shopping activity duration, there is no right censoring because all the individuals end their shopping activities in the observation period [2]. Thus, in the case of shopping activity duration, the likelihood function for the MAFT model can be simplified as:

$$L=\prod_{i=1}^{N}f(T_{i}|U_{i},V_{i})$$ (26)

With the natural logarithm function ln(∙), the log-likelihood function to be maximized can be formulated as:

$$LL=\sum_{i=1}^{N}ln[f(T_{i}|U_{i},V_{i})]$$ (27)

### 2.4 The goodness of model fit

For the goodness of model fit, many statistical measures can be used, such as log-likelihood value, Chi-square statistic and adjusted ρ^2^ value, etc. And the likelihood ratio test, Akaike information criteria (AIC) and Bayesian information criterion (BIC) are commonly considered to compare overall goodness-of-fit among different duration models. The values of AIC and BIC can be calculated based on the following equations:

AIC=2(K−LL) (28)

BIC=−LL+0.5∙K∙ln(N) (29)

where *LL* is the log-likelihood value at convergence and *K* represents the number of estimated parameters in the model.

Nevertheless, in this study, the MAFT model and the TAFT model cannot be compared using the standard likelihood ratio test because they belong to non-nested structures, where no model can be achieved by simply restricting parameter(s) in another model. Cox [30] proposed a statistical test to compare the models based on separate families of hypotheses. Horowitz [31] simplified this test to make it more applicable in the context of discrete choice models but this non-nested test can also be applied to compare the MAFT model and the TAFT model in this study. For non-nested test application, the following statistics need to be first computed:

$${{\bar{\rho }}_{m}}^{2}=1-\frac{{LL}_{m}-K_{m}/2}{LL^{*}}$$ (30)

$${{\bar{\rho }}_{t}}^{2}=1-\frac{LL_{t}-K_{t}/2}{{LL}^{*}}$$ (31)

where ${{\bar{\rho }}_{m}}^{2}$ and ${{\bar{\rho }}_{t}}^{2}$ are the adjust *ρ*^2^ for the MAFT model and the TAFT model; *LL*_*m*_ and *LL*_*t*_ are log-likelihood function values for the models respectively; *K*_*m*_ and *K*_*t*_ are the number of estimated parameters in the two models respectively; LL* is the log-likelihood function value of the model without any explanatory variables.

Since *K*_*m*_ equals *K*_*t*_ in the two models, then

$${{\bar{\rho }}_{m}}^{2}-{{\bar{\rho }}_{t}}^{2}=\frac{LL_{t}-{LL}_{m}}{{LL}^{*}}$$ (32)

The non-nested test statistic is

$$P({{\bar{\rho }}_{m}}^{2}-{{\bar{\rho }}_{t}}^{2}>z)\leq \Phi (-\sqrt{-2{LL}^{*}z})$$ (33)

where Φ() represents the cumulative distribution function of standard normality and z takes a positive value. If the adjusted likelihood ratio index of the MAFT model is greater by some z>0 than the TAFT model and the model with greater adjusted likelihood ratio index is selected, the right-hand side of the Eq (33) bounds the probability of erroneously selecting the incorrect model. Thus, the non-nested test can be applied to select a better fitted model between the MAFT model and the TAFT model.

## 3. Data

### 3.1 Data source

The dataset used for this study was derived from the 2017 National Household Travel Survey (NHTS). The NHTS is the inventory of the nation’s daily travel behavior and conducted by the U.S. Department of Transportation (USDOT). The NHTS data are collected directly from a stratified random sample of U.S. households.

The survey includes four main parts: demographic characteristics of households, people, vehicles, and detailed information on daily travel for all purposes by all modes. Every household, person and vehicle has unique identifier. The daily trip data include an inventory of all trips made on the assigned travel date by all household members aged 5 or older. The designed 24-hour travel day started at 4:00 a.m. (local time) of the assigned travel day and ended at 3:59 a.m. of the following day. 4:00 a.m. represents a time when a relatively small number of people are traveling. Starting the travel day at this time increased the likelihood that most household members would be at home at the start of their travel day. For each trip, respondents report trip purpose (trip origin purpose and trip destination purpose), mode of transportation, beginning and ending time of the trip, travel day of the week and number of people together. These data can be linked with respondents’ demographic characteristics (gender, age, driver and worker status, etc.), household socio-economic characteristics (income, number of workers, housing type, and home location), demographic characteristics of other members in the household and the household vehicle characteristics (model and year).

### 3.2 Sample description

The shopping activity sample used for analysis and model estimation was extracted from the survey data after a series of data assembly and screening. This paper focuses on the grocery shopping activity made by commuters of Texas after work. The final sample consists of 2092 grocery shopping activities, undertaken by 2092 commuters after work. This means that every commuter has one grocery shopping trip after work. “State FIPS for household address” can be used as the indicator to select trip data of Texas. Commuters are the travelers who make at least one work trip on the survey day. To extract shopping activities from the trip diary, trip purpose is considered as the main indicator. A grocery shopping trip has a destination purpose of grocery shopping, and the grocery shopping activity continues from the ending time of this grocery shopping trip to the beginning time of the next trip. Grocery shopping activity after work can be determined by trip number. If the trip number of a grocery shopping trip is larger than that of the last work trip, this grocery shopping trip and subsequent grocery shopping activity can be added into the sample.

Table 1 presents descriptive statistics of explanatory variables selected for the model and their definitions. Work characteristics are important variables affecting the duration of shopping and other non-work activities. Four main work characteristics are included: work duration, travel time to work, departure time from work before 5:30pm and mode to work being motorcycle. The last two work characteristics are dummy variables.

**Table 1:** Variable definitions and descriptive statistics.

Variable	Definition	Descriptive Statistic
**Dependent Variable**		
Shopping duration	Duration of shopping activities (min)	28.47
**Work characteristics**		
Work duration	Time between arrival at work in the morning to departure from work in the evening (min)	456.96
Departure time from work before 5:30pm	1 if individual departs from work before 5:30 pm, 0 otherwise	75.2%
Travel time to work	Travel time to work without traffic (min)	30.33
Mode to work: motorcycle	1 if motorcycle is the mode to work, 0 otherwise	0.3%
**Shopping characteristics**		
Number of females shopping together	Number of females shopping together (including the respondent)	0.68
0		38.4%
1		56.4%
2		4.6%
3+		0.6%
Wednesday	1 if the shopping trip is made on Wednesday, 0 otherwise	18.6%
**Socio-demographic characteristics**		
Household with one adult, child(ren) aged 0–5 years	1 if Household with only one adult and child(ren) aged 0–5 years, 0 otherwise	0.5%
Household income less than $14,999	1 if Household income less than $14,999, 0 otherwise	4.6%
Not in MSA (Metropolitan Statistical Area)	1 if the household's home address is not in the MSA (Metropolitan Statistical Area), 0 otherwise	6.4%
Population density less than 1,999 persons per square mile	1 if population density (persons per square mile) in the census block group of the household's home location is less than 1999, 0 otherwise	36.1%

## 4. Empirical results

### 4.1 Test for proportional hazard assumption

**Fig 1:**
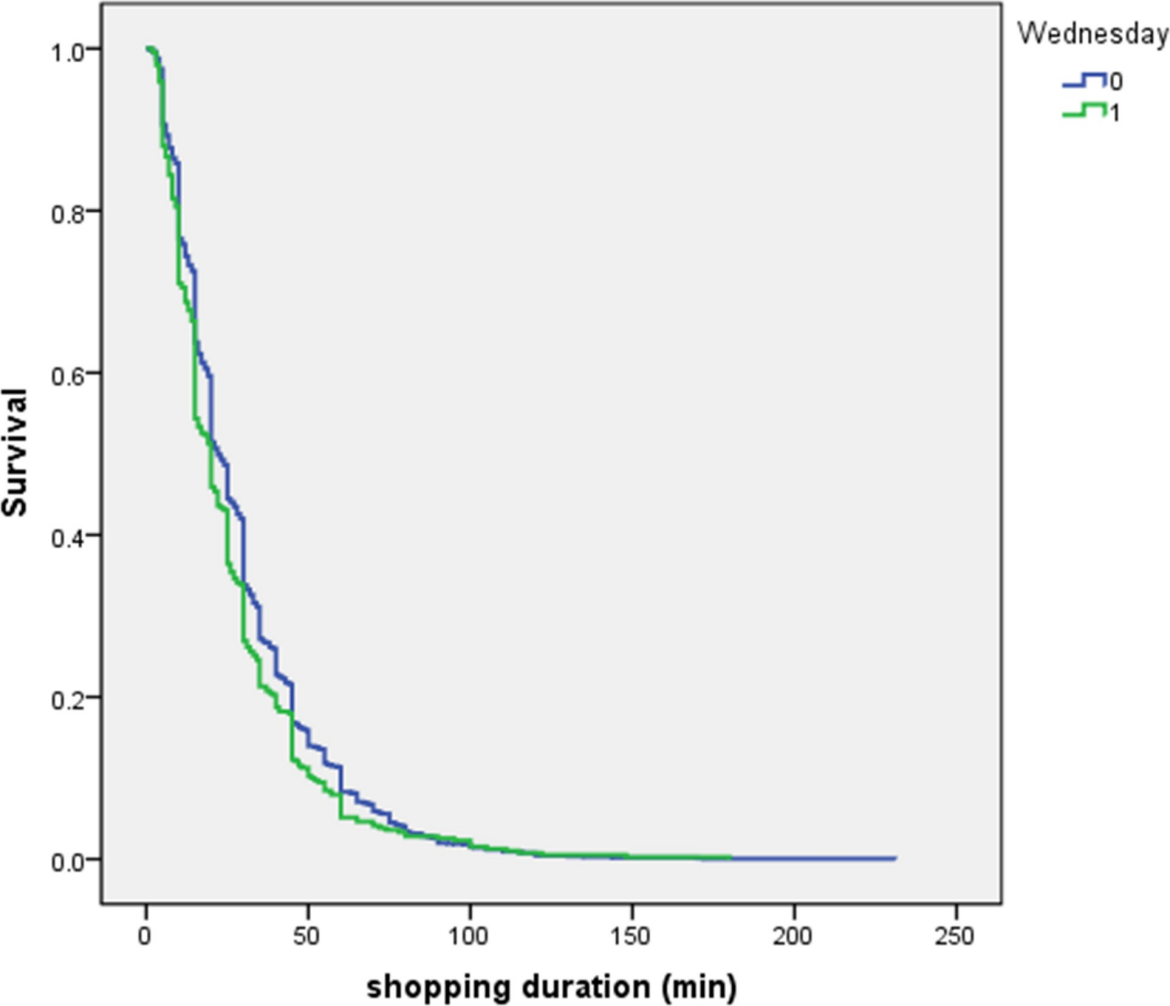
Kaplan-Meier curves (survival—shopping duration).

**Fig 2:**
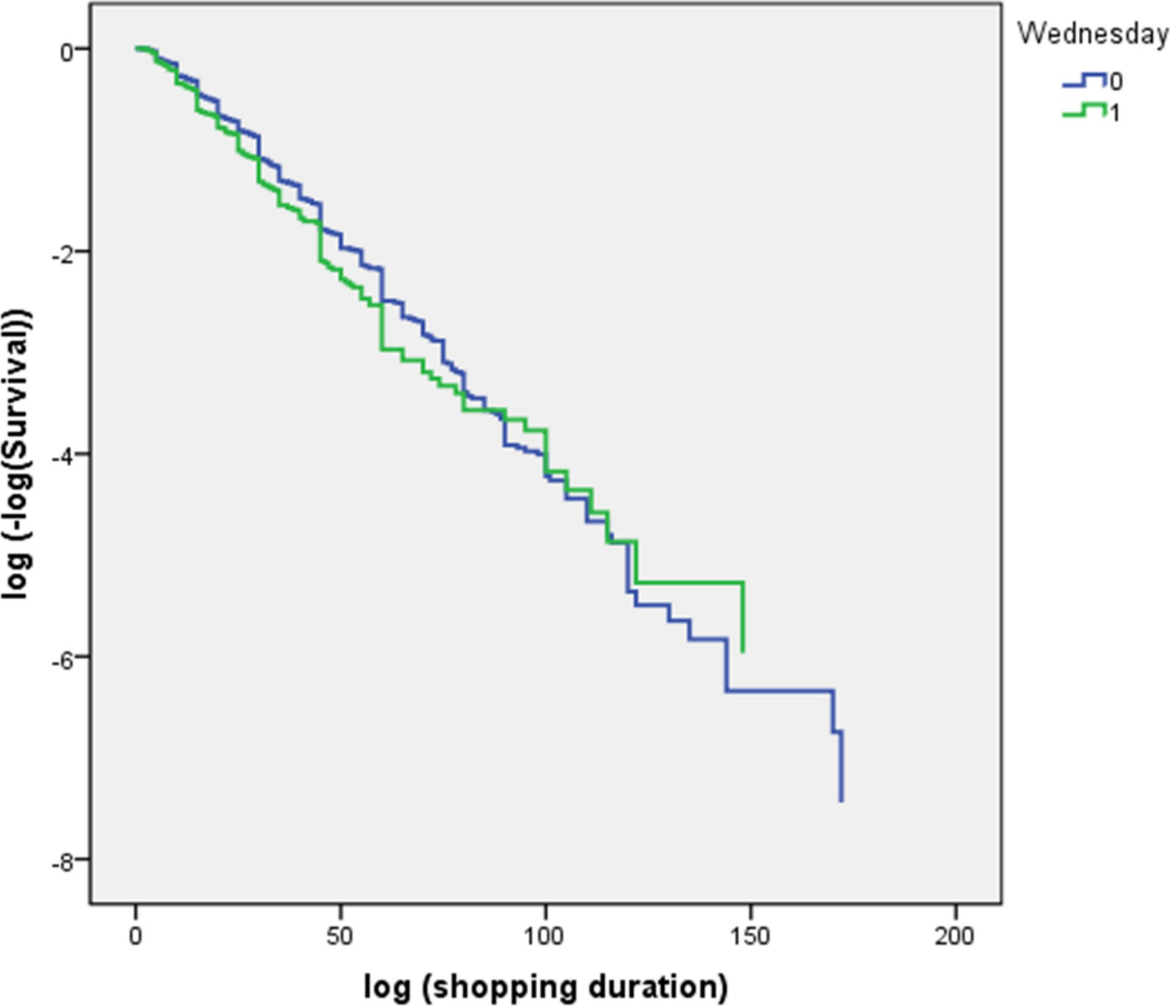
Kaplan-Meier curves (log(-log(survival)–log(shopping duration)).

Proportionality assumption is one of critical assumptions in PH models. However, the assumption of proportionality is untenable in most cases and should be tested before a PH model is applied. First, Kaplan-Meier curves can be used to test the proportionality assumption. Kaplan-Meier curves were plotted and shown in Fig 1 and Fig 2 when “Wednesday” was put as the chosen dummy covariate. The two curves in each of the two figures are not parallel, which indicates that the proportionality assumption is violated.

The test for the validity of the proportionality assumption can also be based on the Schoenfeld residuals [32]. The test can be carried out by adding time-dependent variables to the original PH model. Proportionality assumption is valid only if covariates in the model are time independent [9]. If the time-dependent specification is statistically significant, then there is an evidence showing that the proportionality assumption is violated. The time-dependent variables can be calculated as the interactions between the covariate and a function of survival time, typically the natural log of survival time. For a proportionality assumption test, overall goodness-of-fit between two Cox models are compared.

The test results show that some interactions are significant statistically, for example “the number of females together”. Thus, it indicates that the proportionality assumption is not valid for the sample data used in this study with the current explanatory variables. The Chi-square statistic is calculated to compare the overall goodness-of-fit between the two Cox models with a value of 639.29 for 11 degrees of freedom, indicating that two Cox models have a difference at a significance level of 0.01. Since the Cox model with time-dependent covariates performs better than the original Cox PH model, it evidences again that the proportionality assumption is untenable in this analysis. In other words, the PH model should not be applied in this analysis with given factors for the shopping activity duration, and the TAFT model can be selected as an alternative. Based on the consideration that not all the covariates are time-dependent, the MAFT model can be applied instead of the TAFT model and helps to find the best fitted AFT model.

### 4.2 Empirical estimation results

For comparisons between the MAFT model and the TAFT model, the estimation results of the two models are presented in this section. The commonly used distributions for the hazard function, including Weibull, log-logistic, lognormal, Gamma, Gompertz and general F distribution, are explored and similar model estimation results are obtained. In the interest of brevity, Table 2 displays the empirical results of models based on the log-logistic baseline hazard distribution which exhibits the best statistical performance.

**Table 2:** Comparison between the MAFT model and the TAFT model.

Explanatory Variables	the TAFT model		the MAFT model				non-nested test
	X estimation	t-Statistic	U estimation	t-Statistic	V estimation	t-Statistic	
**Work characteristics**							
Work duration/100	-0.041	-3.342	-0.098	-6.119	--	--	7.215E-13
Departure time from work before 5:30pm	0.105	2.416	--	--	0.078	1.945	--
Travel time to work/100	-0.134	-1.976	-0.261	-3.183	--	--	2.070E-04
Mode to work: motorcycle	-0.880	-2.742	--	--	-0.834	-3.160	--
**Shopping characteristics**							
Number of females together	0.245	8.001	--	--	0.2517	8.855	--
Wednesday	-0.157	-3.437	--	--	-0.1566	-3.618	--
**Socio-demographic characteristics**							
Household with one adult, child(ren) aged 0–5 years	-0.390	-1.427	--	--	-0.301	-1.716	--
Household income less than $14,999	0.183	2.076	--	--	0.203	2.675	--
Not in MSA (Metropolitan Statistical Area)	-0.239	-3.147	--	--	-0.244	-3.347	--
Population density less than 1,999 persons per square mile	-0.121	-3.142	--	--	-0.119	-3.219	--
**Auxiliary parameters**							
scale parameter	0.043	11.638	0.037	13.059			
shape parameter	2.155	55.347	1.890	37.493			
**Goodness-of-fit measures**							
ln(L)	-8890.226		-8862.759				
AIC	17806.452		17749.518				
BIC	8939.924		8908.634				
non-nested test	--		6.232E-14				

Based on the log-logistic baseline hazard distribution, the likelihood function for the MAFT model can be written as:

$$L=\prod_{i=1}^{N}f\left(T_{i}|U_{i},V_{i}\right)=\prod_{i=1}^{N}\;e^{-V_{i}\alpha_{i}-U_{i}\theta_{i}}\cdot f_{0}\left(T_{i}\cdot e^{-V_{i}\alpha_{i}}\right)\cdot {\left[S_{0}\left(T_{i}\cdot e^{-V_{i}\alpha_{i}}\right)\right]}^{\exp\left(-U_{i}\theta_{i}\right)-1}=\prod_{i=1}^{N}\;e^{-V_{i}\alpha_{i}-U_{i}\theta_{i}}\cdot \lambda \cdot p\cdot {\left(\lambda \cdot T_{i}\cdot e^{-V_{i}\alpha_{i}}\right)}^{p-1}\cdot {\left[1+{\left(\lambda \cdot T_{i}\cdot e^{-V_{i}\alpha_{i}}\right)}^{p}\right]}^{-\exp\left(-U_{i}\theta_{i}\right)-1}$$ (34)

where *λ* and *p* are scale and shape parameters of the log-logistic distribution respectively.

As a first step, the TAFT model with the log-logistic baseline hazard distribution was developed. The estimation results of the TAFT model are presented on the left of Table 2. For the MAFT model, the different effects of covariates on the hazard function need to be captured. Each covariate was examined sequentially under two different situations as discussed in Subsection 2.2. The model with greater log-likelihood value at convergence and reasonable t-test values is considered as a candidate and then the non-nested test is employed to examine the significance and finalize the specification. The model estimation results of the MAFT model are displayed on the right of Table 2.

It is noted that “X estimation” in Table 2 means the estimation of covariates in the TAFT model. For the MAFT model, “U estimation” represents estimation results for covariates in the first situation, as discussed in Subsection 2.2, which have proportional and time-independent effects on the hazard. Thus, coefficients in the corresponding “V estimation” remain blank. “V estimation” represents estimation results for covariates in the second situation, as discussed in Subsection 2.2. Those covariates interact with time directly in the baseline hazard in the MAFT model and do not have additional proportional effect on the hazard value, therefore there is a blank in the corresponding “U estimation”.

#### 4.2.1 The goodness-of-fit of the empirical models

The better fitted model between the TAFT and MAFT models is selected based on AIC value, BIC value and the non-nested test value. The log-likelihood value at convergence for the TAFT model is -8890.226 while the corresponding value for the MAFT model is -8862.759. Thus, the MAFT model shows an improvement in the model goodness-of-fit. Since the two AFT models are mutually non-nested, a non-nested test was applied and the probability value from the test statistic is 6.232E-14, meaning that the MAFT model performs significantly better than the TAFT model.

If the two AFT models are compared, the two covariates including “work duration” and “travel time to work” have different effects on the hazard function. To differentiate the contribution of those two covariates in the final MAFT model, the non-nested test was undertaken separately. The result indicates that the two covariates significantly improve the MAFT model. This statistical result evidences that the variables “work duration” and “travel time to work” do only have a proportional effect but has no time-dependent effect on the hazard function. In summary, with contributions from the more flexible specification, the MAFT model fits the post-work grocery shopping activity data significantly better than the TAFT model, as evidenced by the lower BIC and AIC values and a final non-nested test for the overall model performance.

#### 4.2.2 Covariate effects

The effects of covariates for the final MAFT model are presented on the right of Table 2. They are classified into work-related characteristics, shopping-related characteristics and socio-demographic characteristics. A positive coefficient implies a longer duration or a lower hazard.

**Fig 3:**
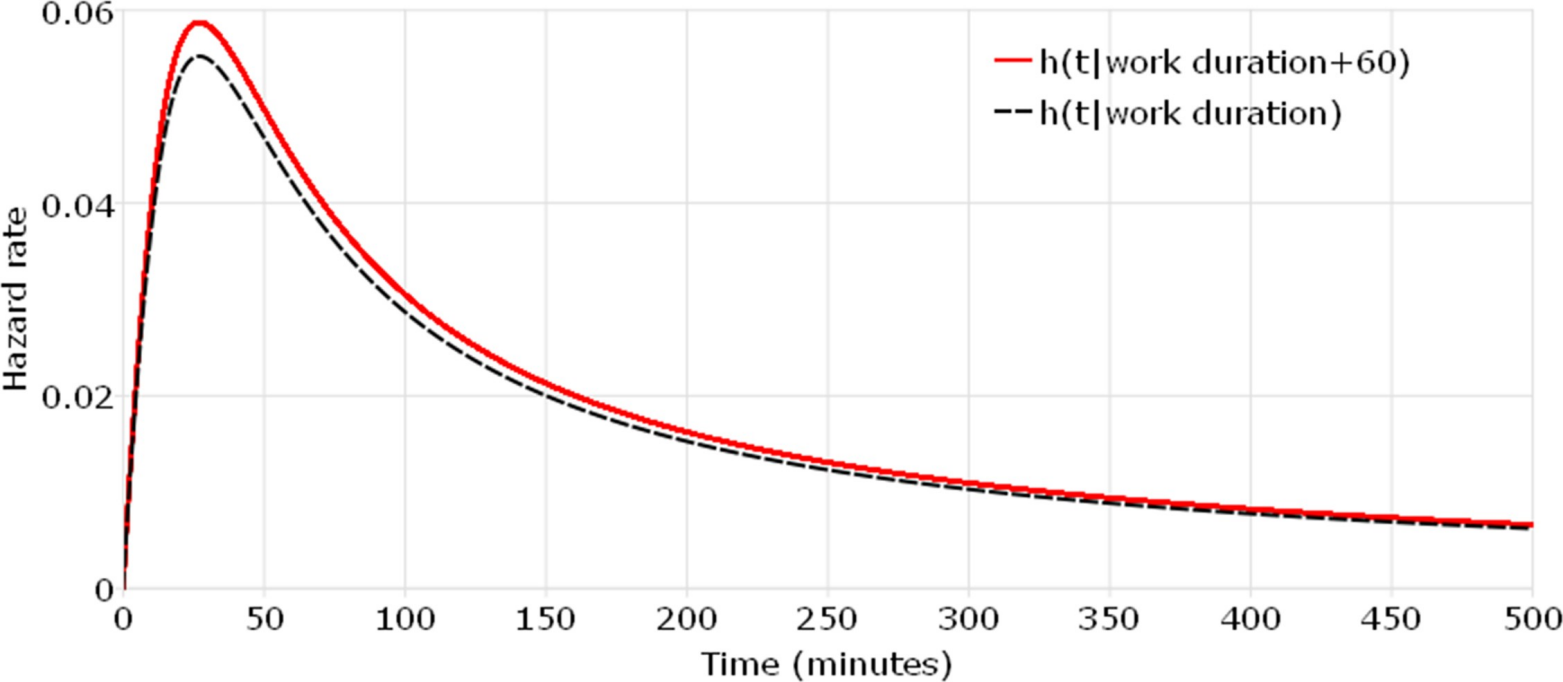
Comparison of hazard rates when work duration increases 60 minutes (work duration in the MAFT model).

**Fig 4:**
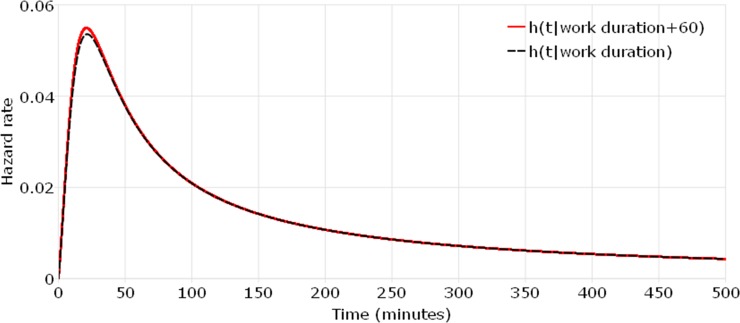
Comparison of hazard rates when work duration increases 60 minutes (work duration in the TAFT model).

Differing from that all covariates have both time-dependent and non-proportional effects on the hazard function in the TAFT model, the empirical MAFT model captures two covariates with different effects on the hazard function. “Work duration” and “travel time to work” have proportional and time-independent effects on the hazard function, which is the same as covariates in a PH model. These two key work-related covariates are determined prior to shopping activities and only increase/decrease shopping durations proportionally among individuals and the effect does not change over time. For better understanding the difference between MAFT model and TAFT model, the hazard rates are plotted and compared when work duration increases by 60 minutes. For a commuter who works for 480 minutes, leaves work at 5pm and spends 30 minutes on travel between work place and home, Fig 3 presents a comparison between h(t|*work duration* + 60) and h(t|*work duration*) based on the MAFT model. A similar comparison based on the TAFT model is shown in Fig 4. The hazard ratio in Fig 3 is constant over time, which equals $e^{-\Delta work\;duration∙\beta_{work\;duraion}}$. However, the ratio of two hazard rates in Fig 4 equals $e^{-\Delta work\;duration∙\beta_{work\;duraion}}∙\frac{h_{0}(t\;e^{-(work\;duration+\Delta work\;duration)∙\beta_{work\;duraion}})}{h_{0}(t\;e^{-work\;duration∙\beta_{work\;duraion}})}$, which is time-dependent. The contrast between Fig 3 and Fig 4 shows that the TAFT model do underestimate the effect of work duration on the shopping duration when this key covariate is forced to be time-dependent in the model but it is actually not. The MAFT model can accommodate both time-dependent and time-independent covariates and therefore allows the “work duration” covariate to have a proportional effect only.

All the explanatory variables take coefficients with reasonable signs. There are four work-related characteristics: “work duration”, “departure time from work before 5:30 pm”, “travel time to work” and “mode to work: motorcycle”. Consistent with previous research [2,4,7], work duration negatively affects the duration of shopping. This indicates that individuals, who allocate more time on work, have less time available for shopping. “Departure time from work before 5:30 pm” has a positive effect on shopping duration presumably because that the earlier departure time from work will leave more spare time for shopping activities and enough time to go back home. The earlier departure time from work may also induce individuals to spend more time on shopping than initially planned. The longer it takes to travel to work, the shorter the time is available for shopping. Thus, “travel time to work” has a negative sign in the models. There is a negative relationship between “mode to work: motorcycle” and shopping duration. It indicates that commuters who travel to work using motorcycles usually have shorter shopping duration probably because the motorcycle is not convenient to carry many items.

The shopping-related characteristics include “number of females together” and travel day of week-“Wednesday”. The “number of females together” has a significantly positive effect on the shopping duration in the final MAFT model. More people shopping together, the longer shopping duration[4,33]. Especially for female shoppers, a single male usually has a shorter shopping duration than a single female. More female shoppers together tend to discuss with each other and ask for advice from other female shoppers together. And they may consider buying the same thing when one decides to buy something. Thus, more females shopping together tend to generate more demand for shopping and therefore lengthen shopping duration. Commuters’ shopping duration in the mid of a week will be relatively short, as shown by the negative coefficient of the covariate “Wednesday”. It is possibly because a grocery shopping activity is usually undertaken on or near weekends and those occurring in the mid of a week tend to be casual and shorter.

Among socioeconomic and demographic characteristics, several behaviorally intuitive covariates can be discerned. A “household with one adult, child(ren) aged 0–5 years” has a negative effect on the shopping duration. There is little available time for commuters in such households to undertake shopping activities. Single parents need to spend more time to take care of young children after work. “Household income less than $14,999” has a positive sign in the models. The reason behind may be that commuters in lower income households have lower time values and tend to spend more time on comparing cost-effective goods. Since commuters living in an area with lower population density usually have easier access to shopping stores, they may prefer to undertake more frequent but shorter shopping activities. For the same reason, “Not in MSA” and “population density less than 1,999 persons per square mile” both have negative signs in the models.

Auxiliary parameters of log-logistic distribution are shown at the bottom of Table 2. It is observed that the log-logistic distribution’s shape parameter, p, is greater than 1, implying a non-monotonic baseline hazard, as illustrated in Figs 3 and 4.

## 5. Conclusions and discussions

This paper generalizes a TAFT model to accommodate both time-dependent and time-independent covariates in one model, called a MAFT model. The primary objective of this model is to differentiate situations where covariates may have either time-dependent or time-independent effect on the hazard rate for modeling activity duration. Most of previous shopping duration models were developed based on the PH model. Once the proportionality assumption is violated, the AFT model is selected as an alternative. However, a traditional AFT model only has covariates with non-proportional and time-dependent effects on the hazard overtime while a PH model only accommodates covariates with proportional and time-independent effects. The MAFT model developed in this study can accommodate various types of covariates, some of which may have proportional and time-independent effect, some may have non-proportional and time-dependent effects on the hazard value in the same model. A non-nested test can be applied to compare the goodness of model fit between the TAFT and MAFT models and finalize specifications.

The MAFT model is applied to model post-work grocery shopping activity duration in Texas, US. Checking on the proportionality assumption indicated that that assumption is violated. Then, a TAFT model and MAFT model are estimated to analyze influential factors of shopping duration. Model estimation results show that there do exist two different types of covariates significantly affecting shopping activity duration, including covariates only with proportional and time-independent effects, and those with both non-proportional and time-dependent effects. Two key work-related covariates including “work duration” and “travel time to work” have time-independent and proportional effects on the hazard function, which are the same as covariates in a PH model. All the other factors interact with time in the baseline hazard without an additional proportional effect on the hazard function, which are the same as covariates in a TAFT model. The key finding of this paper indicates that it is critical to develop a flexible duration model with both time-dependent and time-independent covariates for accurately evaluating travel demand management (TDM) policies, like flexible work hours, which may affect non-work activity (e.g. shopping) duration of commuters. Otherwise, the impact of work hours on non-work activity duration may be seriously underestimated, as shown in this study.

In future studies, more complex MAFT models may be explored in order to extend the MAFT model and make it even more flexible for modeling activity duration. Unobserved heterogeneity may be added into MAFT models. Van den Berg et al. (2012) and Karimi (2013) adopted the latent class method to capture the heterogeneity across population in the TAFT model [9,13]. Hasan et al. (2013) developed a random-parameter TAFT model to deal with the unobserved heterogeneity [34]. Other similar modeling methods [35–38] can also be explored to capture heterogeneities in a MAFT model in future research.

## Supporting information

S1 Data Data. (SAV)

S1 Fig Kaplan-Meier curves (Survival—shopping duration). (TIF)

S2 Fig Kaplan-Meier curves (log(-log(Survival)–log(shopping duration)). (TIF)

S3 Fig Comparison of hazard rates when work duration increases 60 minutes (work duration in the MAFT model). (TIF)

S4 Fig Comparison of hazard rates when work duration increases 60 minutes (work duration in the TAFT model). (TIF)

S1 Table Variable definitions and descriptive statistics. (TIF)

S2 Table Comparison between the MAFT model and the TAFT model. (TIF)
