# Supplementary figures and images for: An empirical analysis of post-work grocery shopping activity duration using modified accelerated failure time model to differentiate time-dependent and time-independent covariates
Source: PLoS One. 2018 Nov 21;13(11):e0207810. doi: 10.1371/journal.pone.0207810 (PMC6249021; doi:10.1371/journal.pone.0207810)

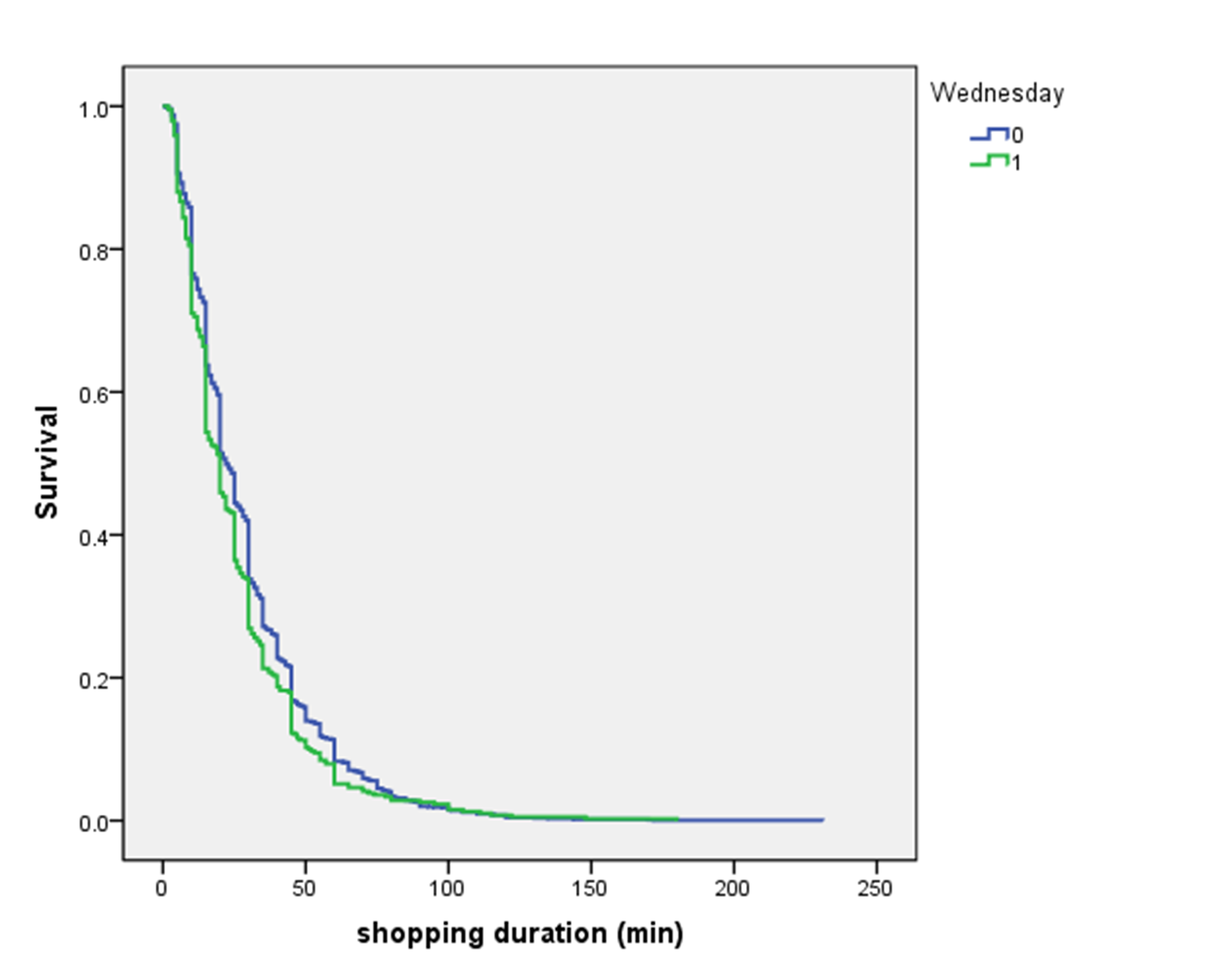

Supplement: S1 Fig — (TIF) [file pone.0207810.s002.tif]

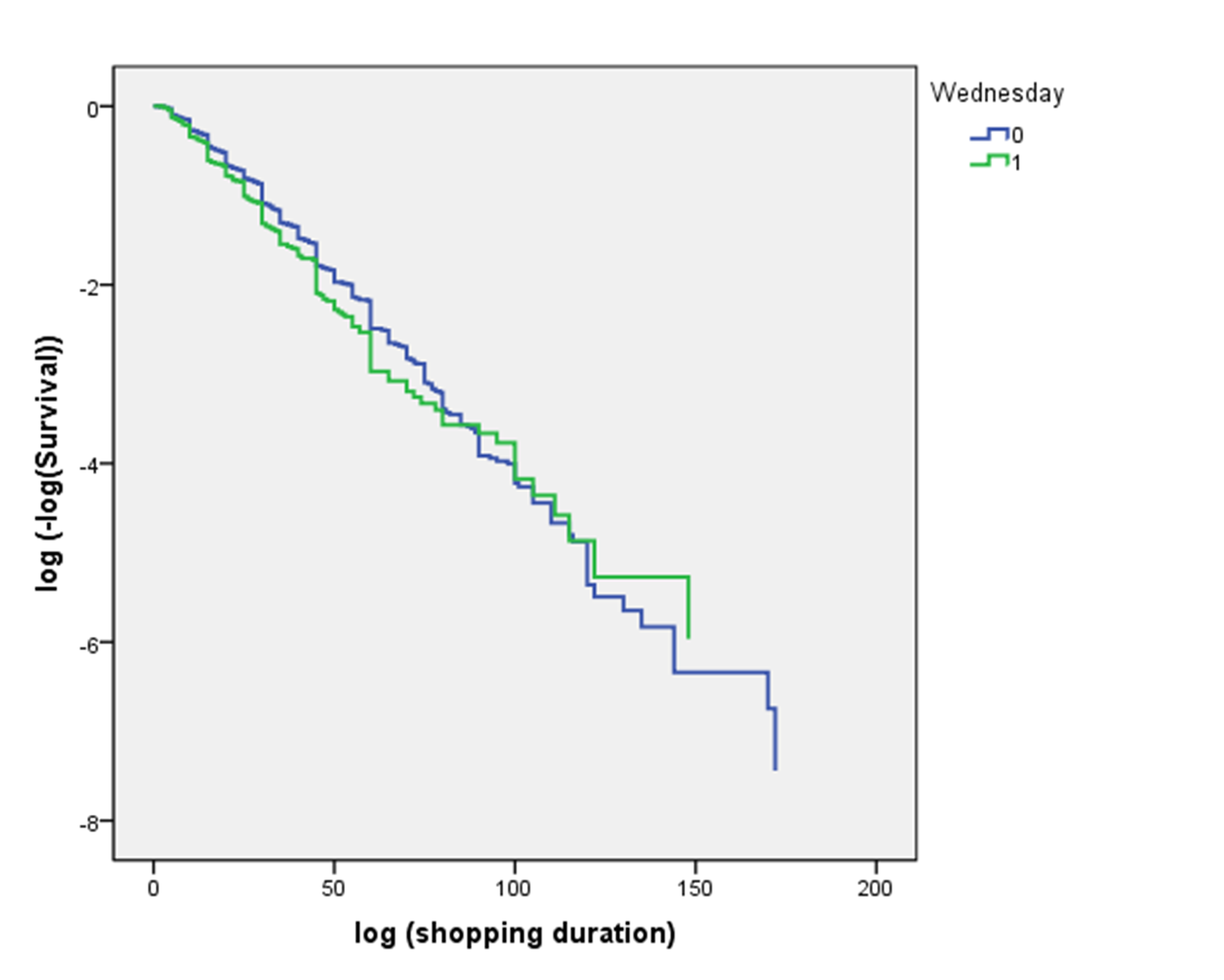

Supplement: S2 Fig — (TIF) [file pone.0207810.s003.tif]

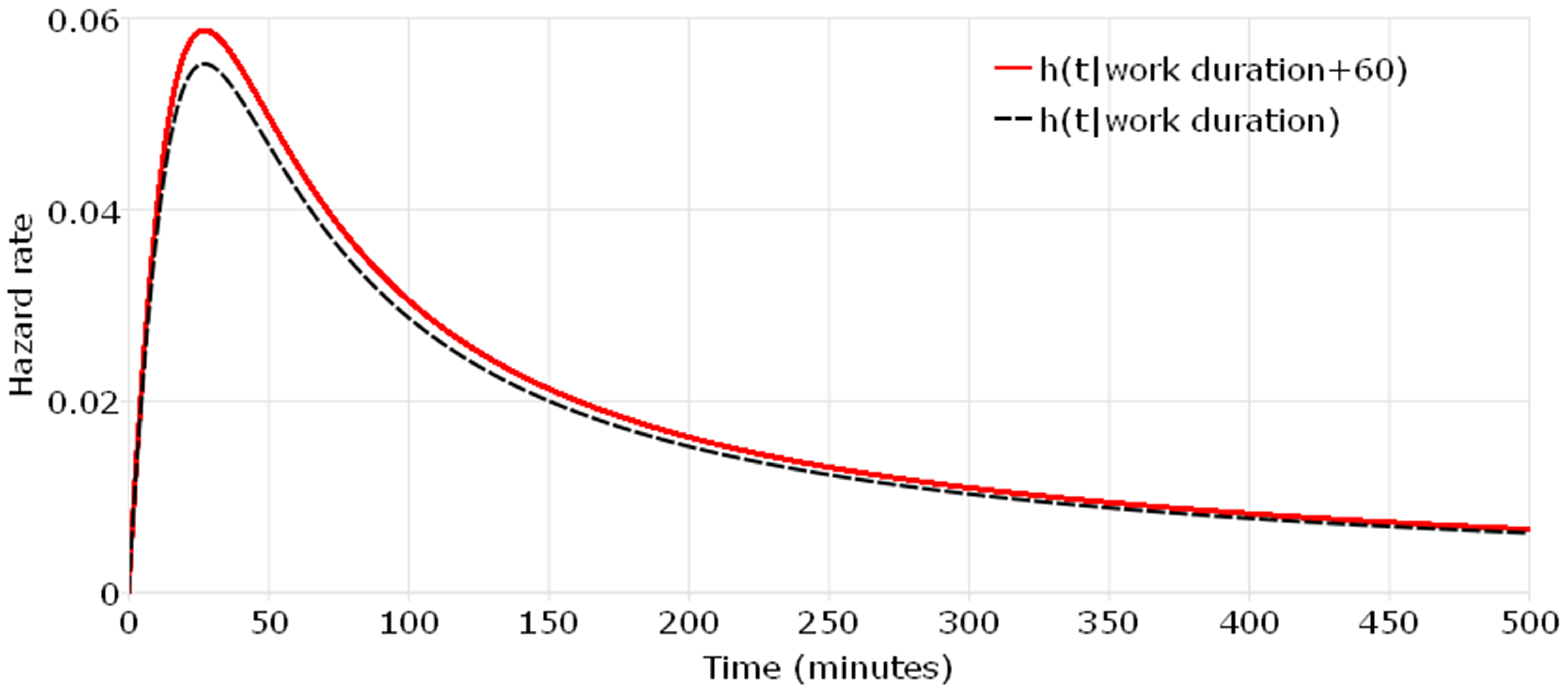

Supplement: S3 Fig — (TIF) [file pone.0207810.s004.tif]

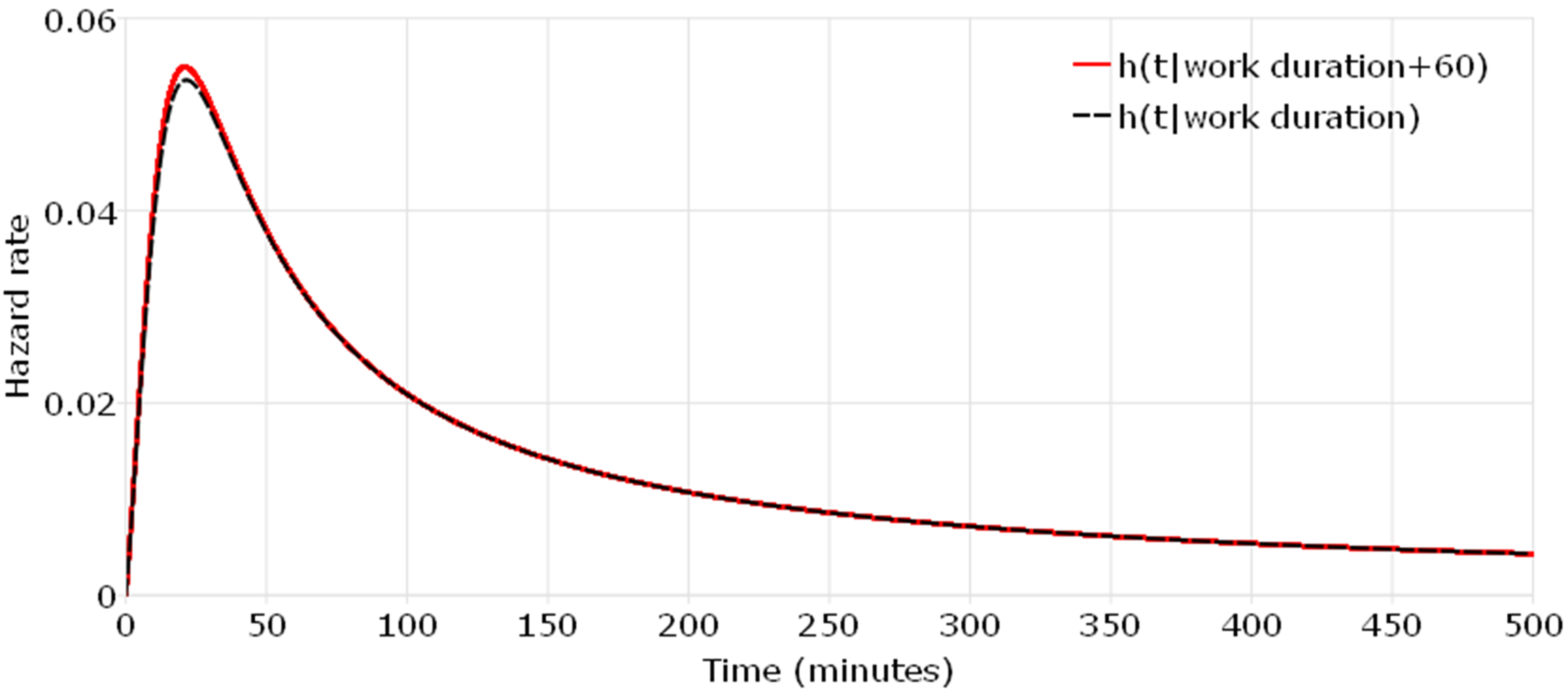

Supplement: S4 Fig — (TIF) [file pone.0207810.s005.tif]

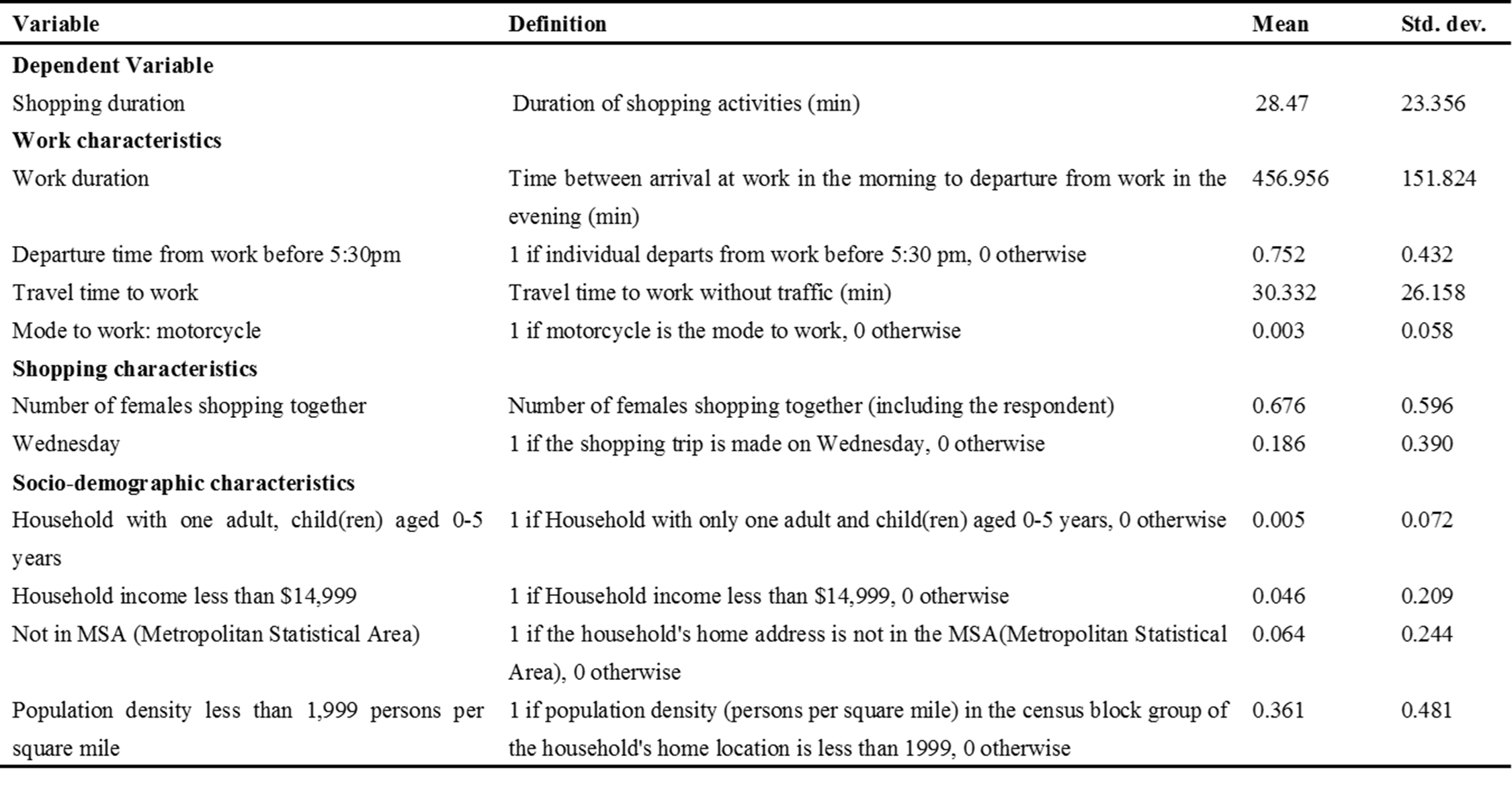

Supplement: S1 Table — (TIF) [file pone.0207810.s006.tif]

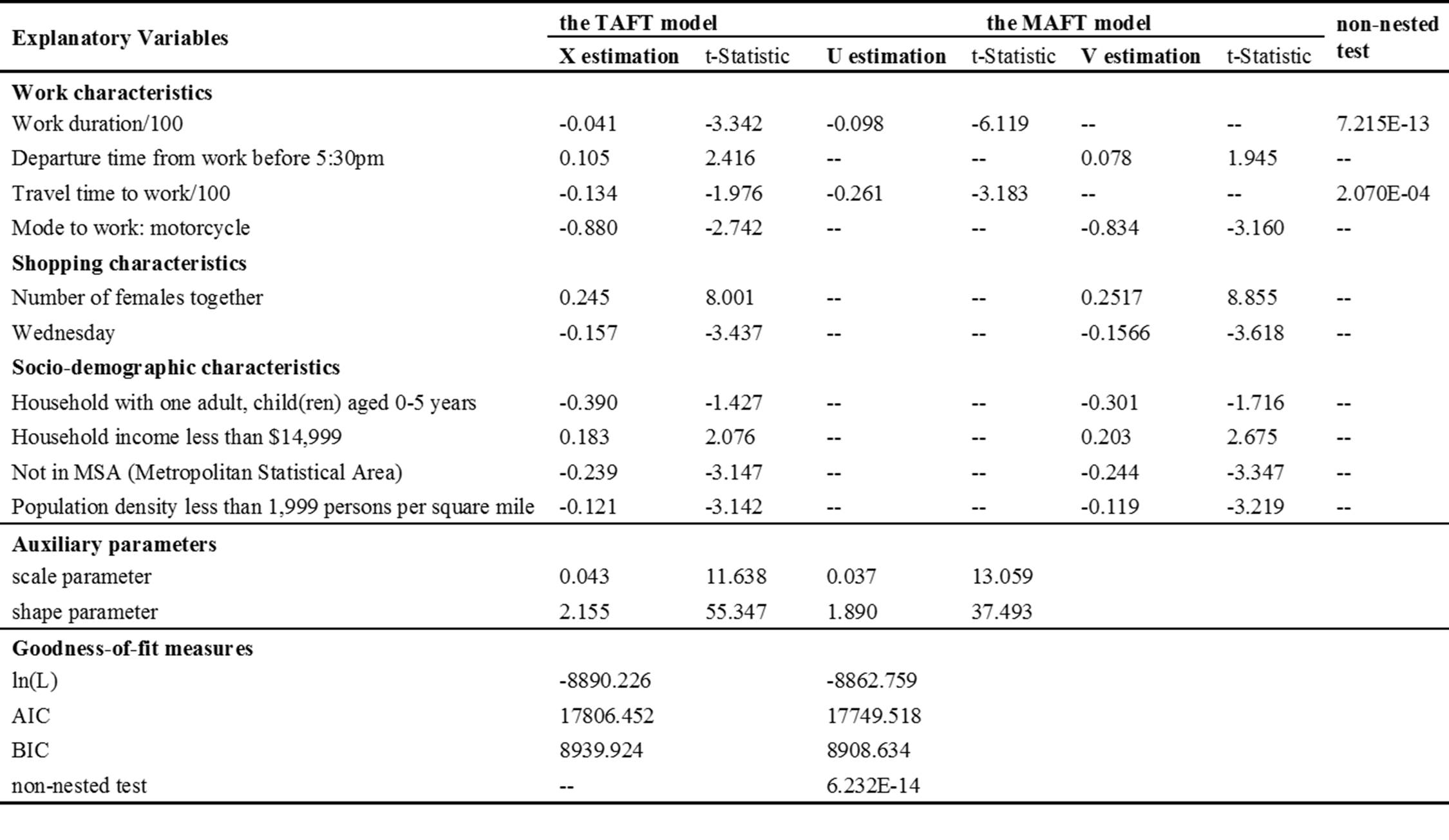

Supplement: S2 Table — (TIF) [file pone.0207810.s007.tif]
